# Quantitative Determination of Cd in Soil Using Laser-Induced Breakdown Spectroscopy in Air and Ar Conditions

**DOI:** 10.3390/molecules23102492

**Published:** 2018-09-28

**Authors:** Xiaodan Liu, Fei Liu, Weihao Huang, Jiyu Peng, Tingting Shen, Yong He

**Affiliations:** 1 College of Biosystems Engineering and Food Science, Zhejiang University, 866 Yuhangtang Road, Hangzhou 310058, China; m15307266704@163.com (X.L.); whhuang1219@163.com (W.H.); jypeng@zju.edu.cn (J.P.); shentingtingstt@163.com (T.S.); yhe@zju.edu.cn (Y.H.); 2 Key Laboratory of Spectroscopy Sensing, Ministry of Agriculture and Rural Affairs, Hangzhou 310058, China

**Keywords:** soil, Cd, laser-induced breakdown spectroscopy, argon

## Abstract

Rapid detection of Cd content in soil is beneficial to the prevention of soil heavy metal pollution. In this study, we aimed at exploring the rapid quantitative detection ability of laser- induced breakdown spectroscopy (LIBS) under the conditions of air and Ar for Cd in soil, and finding a fast and accurate method for quantitative detection of heavy metal elements in soil. Spectral intensity of Cd and system performance under air and Ar conditions were analyzed and compared. The univariate model and multivariate models of partial least-squares regression (PLSR) and least-squares support vector machine (LS-SVM) of Cd under the air and Ar conditions were built, and the LS-SVM model under the Ar condition obtained the best performance. In addition, the principle of influence of Ar on LIBS detection was investigated by analyzing the three-dimensional profile of the ablation crater. The overall results indicated that LIBS combined with LS-SVM under the Ar condition could be a useful tool for the accurate quantitative detection of Cd in soil and could provide reference for environmental monitoring.

## 1. Introduction

Soil is the most important part of the ecosystem, and plays an important role in agricultural production and human activities [1,2]. The rational utilization of soil is beneficial to the healthy and stable development of modern society [3]. However, with the rapid development of industry and agriculture and the frequent use of agricultural chemicals, heavy metals are deposited into the soil through the atmosphere, industrial and municipal domestic sewage, industrial and mining industry solid waste, and agricultural chemicals, making the soil heavy metal pollution more and more serious. Particularly, cadmium (Cd) pollution is one of the most serious soil heavy metal pollutions in China. In recent years, many soil Cd content over standard events have been reported [4,5], which have attracted the public’s attention. Moreover, Cd is one of the most toxic heavy metals, and it is listed as the sixth most hazardous substances by the Agency for Toxic Substances and Disease Registry (ATSDR) and the first carcinogen by the International Agency for Research on Cancer (IARC) [6,7]. Thus, Cd will directly affect and endanger human health when it enters the human body through the food chain, leading many risks such as kidney disease, skeletal damage, and even cancers [8]. Therefore, it is necessary to quantitatively detect the Cd content in soil to monitor environmental quality and ensure food safety.

Currently, atomic absorption spectrometry (AAS) [9], inductively-coupled plasma optical emission spectrometry (ICP-OES) [10], and inductively-coupled plasma mass spectrometry (ICP-MS) [11] are the main methods for the detection of heavy metal in soil. However, these traditional chemical detection methods are limited by the complex detection process, which needs cumbersome sample preparation, and is time-consuming, and environmentally-unfriendly—thus cannot meet the demands of real-time detection.

Laser-induced breakdown spectroscopy (LIBS) is an atomic analysis technique developed in recent years [12]. Specifically, the plasma is firstly produced when the laser ablates the sample, then the qualitative and quantitative determination of elements can be carried out by analysing spectral signals emitted by the plasma [13]. Due to the advantages of fast analytical speed, simple sample pre-treatment, multi-element simultaneous detection, and in-situ detection capability [14], it has been widely used for element analysis in various fields, such as environmental monitoring [15], industrial analysis [16], bio-medicine [17]. However, the high detection limit of elements has always been a difficult problem to restrict the application of LIBS [18]. Therefore, LIBS signal enhancement technologies have become the focus of scholars at home and abroad, mainly including double pulse LIBS [19,20,21], nanoparticle enhanced LIBS [22,23], spatial-confinement LIBS [24,25], resonance-enhanced LIBS [26,27], and so on. Besides, the signal enhancement technique based on argon (Ar) is also one of the most promising techniques for signal enhancement. Chen et al. [28] used Ar as the protective gas to quantitatively detect Mn, Si, and Cr in liquid steel. It was found that Ar could prevent the surface oxidation of molten steel, and enhance the plasma signal while reducing the detection limit of elements. Kim et al. [29] applied LIBS to detect a thin Cu (In, Ga) Se_2_ (CIGS) absorber layer under the conditions of open air and Ar jet flow (25 L min^−1^), and the elements of Cu, In, Ga, and Se were analysed. The results showed that Ar had a great influence on the signal of Se I 196.090 nm, of which the spectral intensity was increased by about three times and the value of RSD decreased by 0.01 (from 0.045 to 0.035). Sarkar used [30] Ar at a flow rate of 6 L min^−1^ to explore the signal enhancement effect of LIBS on uranium (U) in the vitrified simulated high level barium borosilicate waste glass matrix. The results showed that the spectral intensity of U in Ar was about five times higher than that in air. In summary, above studies showed that LIBS detection in Ar could enhance spectral signal, and had great potential for the rapid and accurate detection of elements. To the best of our knowledge, the LIBS technique has been applied to the determination of Cd in water [31], agricultural products [32,33], fertilizers [34], and soils [35]. Besides, the quantitative detection of Cd in soil using the LIBS signal enhancement technique, based on argon (Ar), has also been reported [36], but the influence principle of Ar jet flow has not been investigated.

In this paper, we used the simulated Cd-containing soils as samples to explore the quantitatively detection ability of LIBS for Cd in soil under the conditions of air and Ar, and the enhancement effect of Ar on LIBS signal was further analysed. The specific objectives of this research were to: (1) compare the spectral intensity and system performance under different gas conditions; (2) to build univariate and multivariate quantitative analysis models of Cd in soil under different gas conditions; and (3) to further compare the performance of models to explore the principle of LIBS signal enhancement of Ar.

## 2. Materials and Methods

### 2.1. Soil Samples

In this experiment, Cd-containing soil samples were prepared by adding the heavy metal element into the certified reference material (CRM) of soil (GBW07455), which was provided by National Institute of Metrology, P.R. China. Referring to the national soil heavy metal pollution grade standard of China, CdCl_2_ powder of 99% purity purchased from Aladdin Reagent Shanghai Pure Biochemistry Technology Co. Ltd. (Shanghai, China), was used to prepare different concentrations of CdCl_2_ solution (0.02 mol·L^−1^, 0.04 mol·L^−1^, 0.06 mol·L^−1^, 0.08 mol·L^−1^, 0.10 mol·L^−1^, 0.12 mol·L^−1^, 0.14 mol·L^−1^, 0.16 mol·L^−1^, 0.18 mol·L^−1^, and 0.20 mol·L^−1^) and distilled water was used as the solvent. Specifically, 10 g CRM soil powders were firstly added to the CdCl_2_ solution of different concentrations, and they were mixed evenly by a grinder (JXFSTPRP-48, JX, Shanghai, China). Then, the mixture was dried in the oven at 60 °C for 6 h. After the above steps, Cd-containing soil samples with 10 content gradients were prepared. The content of Cd in the soil samples were 0.22 mg·g^−1^, 0.45 mg·g^−1^, 0.67 mg·g^−1^, 0.90 mg·g^−1^, 1.12 mg·g^−1^, 1.35 mg·g^−1^, 1.57 mg·g^−1^, 1.80 mg·g^−1^, 2.02 mg·g^−1^, and 2.25 mg·g^−1^, respectively. Finally, 0.5 g dried powders from soil samples of each content were weighed, and pressed into tablets with 12 mm in diameter and 2 mm in thickness, using 10-MPa force for 60 s (FY-24, SCJS, Tianjin, China). Five pellets were prepared for each content. Thus, 50 soil tablets were used for LIBS experiments under the conditions of air and Ar, respectively.

### 2.2. Spectral Acquisition 

**Figure 1:**
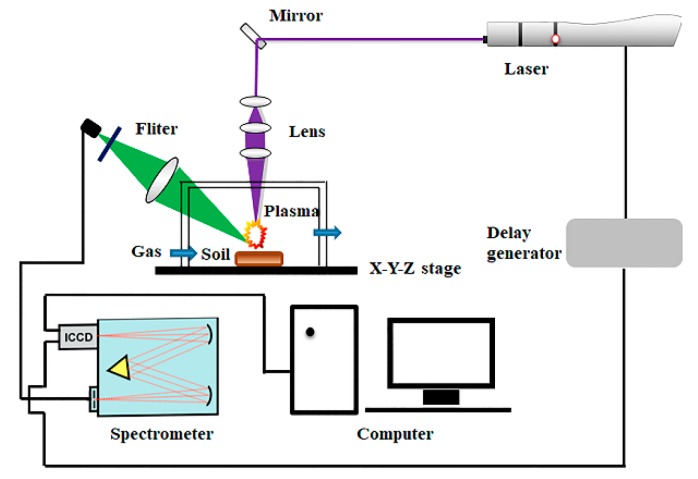
Schematic diagram of LIBS system for soil samples.

A self-built LIBS system used in this experiment is shown in Figure 1. A Q-switched Nd: YAG pulsed laser (Vlite-200, Beamtech Optronics, Beijing, China) was used for ablating samples to generate plasma, with the maximal energy of 200 mJ @ 532 nm, pulse duration of 8 ns and repetition frequency of 1 to 10 Hz. An Echelle spectrograph (SR-500i-A-R, Andor Technology, Belfast, UK) was applied for dispersion of spectral emission lines. An intensified charge-coupled device (ICCD) camera (iStar DH340T, Andor Technology, Belfast, UK) was utilized to detect spectral signal. A digital delay generator (DG645, Stanford Research Systems, San Jose, CA, USA) was used to control the delay time between the laser and the ICCD camera. Lens, mirrors, and other accessories were used to connect the whole optical path. The sample chamber made of quartz glass was used to create an Ar environment. In addition, an Hg: Ar lamp (HG-1, Ocean optics, Winter Park, FL, USA) and a Deuterium Halogen light source (DH-2000-BAL-CAL, Ocean optics, Winter Park, FL, USA) were used to calibrate wavelength and intensity, respectively. Before the experiment, experimental parameters were optimized using the response surface methodology (RSM). As a result, under the condition of Ar, parameters of laser energy, delay time, gate width, and flow rate were optimized at 120 mJ, 4.5 µs, 10 µs, and 6 L·min^−1^, respectively. Parameters under the condition of air were optimized at 120 mJ, 1.827 µs, 10 µs. The experiment was conducted in open air, so flow rate was not considered. After setting parameters, sample tablets were placed on the X-Y-Z stage, and the laser beam was adjusted to focus 2 mm below the sample surface to acquire LIBS spectra. In order to eliminate shot-to-shot fluctuation and obtain reliable datasets, the spectra of 16 spots with 5 times accumulation were collected, and the average of 80 spectra was recorded as the LIBS spectrum for each pellet. The experiment under the open air was done first, and then high purity Ar was passed into the sample chamber through the gas transmission tube to conduct the experiment under the condition of Ar. Instead of vacuuming, we directly passed a steady flow of Ar into the sample chamber for half an hour before the experiment to ensure an Ar atmosphere in the sample chamber. Besides, a steady flow of Ar was continuously passed into the sample chamber during the experiment under the condition of Ar.

### 2.3. Data Analysis

#### 2.3.1. Data Preprocessing

Due to the matrix effect of the sample and the fluctuation of experimental parameters, noise and abnormal spectra might exist in the raw spectra of samples, which might influence the accuracy of data analysis. Thus, spectral pre-processing methods including wavelet transform (WT) and outlier rejection were used to eliminate the influence. WT is a widely used denoising method, which can effectively reduce the noise generated in the experiment [37]. The spectra are decomposed into low-frequency signals and high-frequency signals by WT, and wavelet functions with different decomposition levels will be further analyzed [38]. The optimal wavelet functions and decomposition levels for noise reduction can be selected when the maximal signal-to-noise ratio (SNR) or the minimal root mean square error (RMSE) are obtained. Detailed information can be found in the literature [37]. Daubechies 5 with decomposition scale 3 was optimized in this study. Outlier rejection is a commonly used spectral data processing method, and it has been proved that removing outlier could improve the precision and repeatability of spectral detection [39]. In this work, a self-developed outlier rejection routine was used to remove abnormal spectra based on median absolute deviation (MAD) [40]. In order to meet our demands, the peak intensity of emission Cd 266.5 nm was applied as the variable to identify outliers, as it was relatively stable among the three analysed Cd spectral lines. Specifically, the median and MAD of peak intensity of Cd 266.5 were calculated. Then the spectrum would be regarded as an outlier to be eliminated when the difference between the intensity and median of Cd 266.5 nm was beyond 2.5 times MAD. The routine would loop until there was no outlier or the number of remaining spectra was less than 75% of the original spectra [41].

#### 2.3.2. Quantitative Analysis Methods

Univariate analysis is the most basic elemental quantitative analysis method in LIBS. Under certain conditions, the LIBS spectral intensity is proportional to the content of elements in the sample, as seen in the following equation:

*I* = *αC^d^* (1)

where *I* means the intensity of the spectral line, and *C* means the concentration of elements to be analysed. The *d* means the function related to the element content, and the value of *b* can be 1 when the concentration of the analytical element is low and there is no self-absorption. Therefore, the calibration curve of the element can be fitted according to the intensity of the LIBS spectrum and the concentration of the corresponding element. Then the unknown concentration of the sample can be further calculated by the spectral intensity and the calibration curve. In addition, several indicators are adopted to evaluate the performance of the univariate model. The coefficient of determination for calibration (R^2^_C_) and prediction (R^2^_P_) reflect the prediction accuracy of the model, and the closer the value is to 1, the stronger the prediction ability of the model is. The root mean squared error for calibration (RMSEC) and prediction (RMSEP) reflect the prediction deviation of the model, and the smaller the value is, the better the model prediction performance will be. Besides, the limit of detection (LOD) is also a key indicator of evaluation. According to the definition given by international union of pure and applied chemistry (IUPAC), LOD means the lowest concentration obtained from the minimum analytical signal that can be reasonably detected by a particular analysis step. It reflects the sensitivity of the model, and a lower LOD represents a better detection method. The value could be calculated with the following equation:

*LOD* = (3σ_background_)/*b* (2)

where σ_background_ means the standard deviation of the background intensities, and *b* means the slope of calibration curve.

Partial least-squares regression (PLSR) is a multivariate linear analysis method, and is commonly used for the quantitative analysis of spectra [42,43]. Particularly, this method decomposes the original data linear and mutually independent latent variables by linear transformation, and the first few latent variables containing most information of the original variables will be selected for further analysis [44,45]. In this study, we tried to establish the PLSR model of the Cd concentration in soil and its LIBS signal intensity under different gas conditions using the software of Unscrambler × 10.1. Considering that the model is prone to overfitting when the amount of samples is less than the amount of variables [46], we used full cross-validation to correct the model, and the number of latent variables corresponding to the minimal mean squared error was employed [47]. In addition, evaluation indexes of the PLSR model were the same as those of the univariate model. As for the LOD of the multivariate model, it has recently been discussed in several multivariate techniques [48,49]. Firstly, the sensitivity of the model reflects the fraction of analytical signal with the increase elemental content [50,51], and it could be defined as:

(3) $$SEN=\frac{1}{∥m∥}$$

where *m* is the regression vector of the PLSR model, ∥∥ means the Euclidean norm (vector length) of the *m*. According to Equations (2) and (3), an approximation LOD of the PLSR model could be calculated with the following equation [18,52,53]:

(4) $$LOD=\left(3\sigma_{\mathrm{background}}\right)/SEN\;=\;3\sigma_{\mathrm{background}}∥m∥$$

Least-squares support vector machine (LS-SVM) is an optimized statistical learning method based on the standard SVM [54,55]. Due to the good generalization ability, it has been widely used in solving linear and nonlinear regression problems. In LS-SVM, nonlinear mapping is used to map the original data to a high-dimensional space, in which linear regression is then conducted by linear hyperplane to obtain a fitting function [56,57]. In this work, we employed radial basis function (RBF) as a kernel function to establish the LS-SVM model, because of its good nonlinear solving ability [58]. Optimal parameters sig2 and gam were determined based on a grid-search procedure in the range of 10^3^–10^10^ when the value of RMSECV reached its minimum. The parameter sig2 represents the bandwidth of kernel function. The parameter gam represents the trade-off between the minimum model complexity and the minimum training error [59]. Detailed information of LS-SVM model can be found in these literature [60,61]. In order to avoid the overfitting of the model, full cross-validation was also carried out. In addition, evaluation indicators of the univariate model are also applicable to the LS-SVM model.

### 2.4. Software Tools

Design Expert (ver.8.05, CAMO AS, Oslo, Norway) was employed to design experiments and optimize experimental parameters. Andor SOLIS for Imaging (v4.26, Andor Technology, Belfast, UK) was used to collect LIBS spectra. Unscrambler × 10.1 (CAMO, Process, AS, Oslo, Norway) and MATLAB R2009a (v7.8, The MathWorks, Inc., Natick, MA, USA) was utilized to process data and establish the chemometrics model. In addition, Origin Pro 8.0 SR0 (Origin Lab Corporation, Northampton, MA, USA) was used to draw graphics.

## 3. Results and Discussion

### 3.1. Spectral Analysis

**Figure 2:**
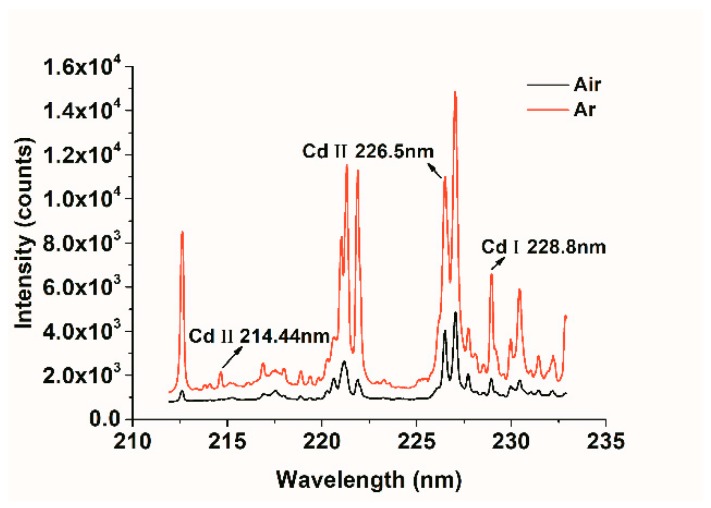
Average spectra of Cd-containing soil samples with medium content in the conditions of air and Argon (Ar).

The spectra were collected over the range from 211.92–232.90 nm, and 2014 variables were contained. According to the National Institute of Standards and Technology (NIST) database, three main characteristic lines of Cd element were identified, including Cd II 214.44 nm, Cd II 226.5 nm, and Cd II 228.8 nm. Due to the consistency of spectral trends among samples with different Cd contents, we only took the sample of medium content (1.12 mg·g^−1^) as an example for analysis. Figure 2 shows the average LIBS spectra under the conditions of air and Ar. It was obvious that the emission intensities of Cd II 226.5 nm and Cd II 228.8 nm were relatively high, and were relatively less affected by the matrix elements. Moreover, it could be seen that the spectral intensities of Cd collected in an Ar environment was increased by about two-to-three times compared with that in air environment. Especially, the peak of Cd II 214.44 nm signal appears only in the collection condition of Ar. The results indicated that Ar was favorable to enhance the element signal of LIBS, and Cd II 226.5 nm and Cd II 228.8 nm were more suitable for further analysis.

**Figure 3:**
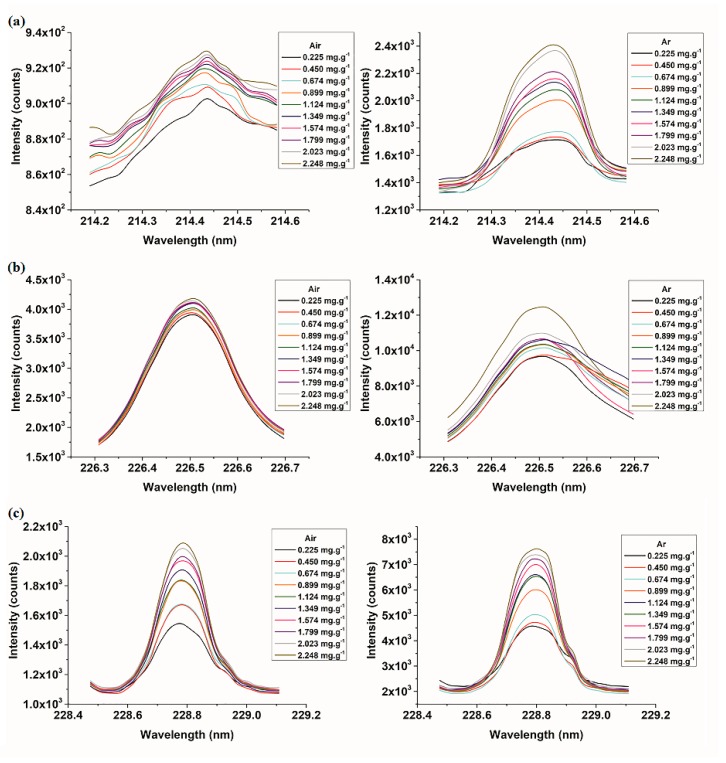
The curve of spectral intensities with Cd contents under the collection conditions of air and Ar. (**a**) Cd II 214.44 nm in air and Ar; (**b**) Cd II 226.5 nm in air and Ar; and (**c**) Cd II 228.8 nm in air and Ar.

Figure 3 shows the curve of the average intensities of three Cd characteristic lines changing with Cd content at 10 content gradients. As seen in Figure 3, the spectral intensities of three Cd characteristic lines increased with the increase of Cd contents roughly, and there was no phenomenon of peak flattening or subsidence caused by self-absorption. Comparing the curve under the collection conditions of air and Ar, we could see that the spectral differences of samples with different Cd contents in Ar were more obvious. Besides, there was a slight shift in the centre position of all the three spectral lines, which might be due to the low resolution of the detector and the slight change of the detector temperature in the experiment. In addition, LIBS quantitative detection of elements would also be disturbed by system noise and an element background signal, so it was necessary to further analyse the systems performance.

### 3.2. System Performance Analysis

**Figure 4:**
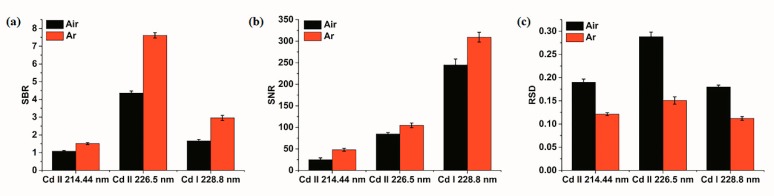
Comparison of system performance under the conditions of air and Ar. (**a**) signal-to-background ratio (SBR); (**b**) signal-to-noise ratio (SNR); (**c**) relative standard deviation (RSD).

Signal-to-background ratio (SBR), signal-to-noise ratio (SNR), and the relative standard deviation (RSD) are important parameters in spectral analysis, which directly reflect the detection ability and stability of the system [62]. SBR is the ratio of net analytical signal to background signal, and SNR is the ratio of net analytical signal to noise interference. RSD is the ratio of standard deviation of signal to average intensity of signal. In detail, the signal intensity was the peak intensity of spectral lines corresponding to the element in the LIBS spectrogram. The background was the spectral line near the spectral line of the element to be analyzed, and the average peak intensity of 20 spectral lines near the spectral line of the element to be analyzed was used as the background intensity in this study. The net analytical signal, used to calculate SBR and SNR, was obtained by subtracting the background from the spectral signal. The level of noise was evaluated by the standard deviation of the background signals. In general, larger values of SNR and SBR represent better detecting ability of the instrument, while the lower value of RSD represents better stability of the system. Therefore, these three parameters were employed to compare the system performance under the conditions of air and Ar. The values of SBR, SNR, and RSD of three Cd characteristic lines for all soil samples, with different Cd contents, were calculated, and it was found that all samples had similar variation trend for these three parameters. Thus, to be consistent with spectral analysis, the sample of medium content (1.12 mg·g^−1^) was taken as an example for analysis. The average value of five samples with a medium content for these three parameters were calculated, and the results are presented in Figure 4.

It was seen that three Cd characteristic lines had a consistent trend. The values of SBR and SNR under the condition of Ar was higher than those in air condition, and a reverse trend was presented by the value of RSD. The results indicated that the detection ability of LIBS and the stability of detection signal under the condition of Ar were superior to those in air condition. Comparing the three spectral lines of Cd, we found that the signal of Cd II 214.44 nm was weak, while the signals of the other two lines were relatively strong, with a high value of SBR in Cd II 226.5 nm and a high value of SNR in Cd II 228.8 nm. However, the value of RSD for Cd II 226.5 nm was large. After a comprehensive comparison, Cd II 228.8 nm was regarded as the most suitable for subsequent analysis.

### 3.3. Quantitative Determination of Cd in Soil Samples

#### 3.3.1. Quantitative Determination of Cd Based on Univariate Models

Before modeling, sample division was firstly conducted. Three samples of each content were assigned for calibration, and the rest was used for prediction. Thus, all soil samples were divided into a calibration set (30 for quantitative analysis under the condition of air and Ar, respectively) and a predication set (20 for quantitative analysis under the condition of air and Ar, respectively) with a ratio close to 2:1. Then, univariate models of peak intensity and Cd content for three Cd spectral lines were established by means of calibration curve. It was found that there was no obvious linear relationship between the spectral intensities of Cd II 214.44 nm and Cd II 226.5 nm and the corresponding Cd concentration in soil samples. The results were similar to the previous study of Li et al. [63,64], which might be due to the matrix effect of soil. Therefore, only Cd II 228.8 nm was selected for analysis in this work. 

**Figure 5:**
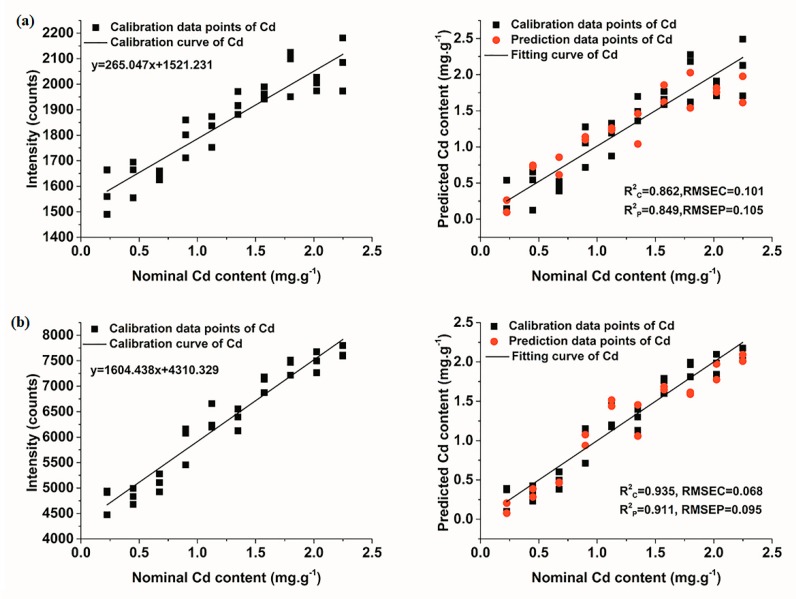
Univariate calibration curves and models of Cd under different conditions (**a**) air; and (**b**) Ar.

Figure 5 shows the results of univariate models of Cd II 228.8 nm. Calibration curves of Cd in Figure 5 were firstly fitted using the calibration set, and the predicted content of Cd could be obtained according to the formula of fitting curve. It was obvious that the performance of univariate model under the condition of Ar was better than that in air condition, with R^2^ in both calibration and prediction sets above 0.9. Besides, the values of RMSEC and RMSEP in the model under the condition of Ar were lower than those in air condition. In addition, according to Equation (2) mentioned above and the slope of calibration curve in Figure 5, the LOD of Cd could be calculated. Under the condition of air, the LOD of Cd in soil samples was 87.1 ± 2.8 ppm, while it was 40.6 ± 0.4 ppm under the condition of Ar. It was apparent that Ar environment could enhance the elemental signal, and meanwhile improve the quantitative detection ability of LIBS for Cd.

#### 3.3.2. Quantitative Determination of Cd Based on Multivariate Models

Due to complex matrix effects of the soil and the rich spectral emission lines of elements, some important information might be lost in univariate analysis, affecting the accuracy of quantitative analysis. In contrast, the multivariate analysis method can effectively reduce the influence of matrix effects, and make full use of the spectral information to improve the accuracy and repeatability of quantitative analysis. Thus, multivariate analysis models including PLSR and LS-SVM, based on the whole collected band (211.92–232.90 nm), were built to quantitatively analyze Cd content in soil samples under the condition of air and Ar. In addition, variables with great contribution in the whole collected data set are conducive to the PLSR and SVM models. Since scores, x and y loadings, and regression coefficient reflect the contribution of the variable, variables with large value of these parameters played an important role in PLSR and SVM analysis. Furthermore, the sample partitioning method was the same as that used in univariate models.

**Figure 6:**
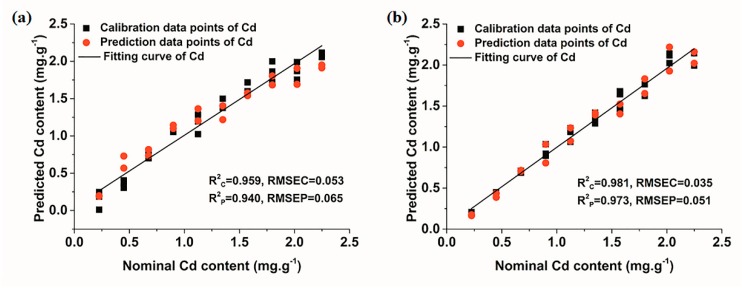
PLSR models of Cd under different conditions. (**a**) air; and (**b**) Ar.

Calibration and prediction results of the PLSR model are given in Figure 6. It was observed that all data points fitted well no matter in the conditions of air or Ar, with all R^2^ being greater than 0.94. Furthermore, it was worth noting that the model performance under the condition of Ar was slightly better than that in air condition, with R^2^ in both calibration and prediction sets over 0.97. Judging from the R^2^ of the model, the enhancement effect of Ar on the quantitative analysis of LIBS was not significant. This might arise from the fact that the quantitative prediction of the model was already very good under the condition of air, so the model performance was more difficult to increase despite the use of Ar for signal enhancement. Besides, according to Equation (4) and the regression vector of the PLSR model that displayed directly in the software of Unscrambler × 10.1, the LOD of PLSR for Cd under the conditions of air and Ar were 14.79 ± 0.47 ppm and 7.17 ± 0.17 ppm, respectively. In terms of detection sensitivity, the enhancement effect of Ar on the quantitative analysis of LIBS was very obvious. The elemental detection sensitivity was reduced by approximately half in the condition of Ar. In general, the results demonstrated the feasibility of quantitative detection of Cd by LIBS coupled with the PLSR model in the condition of Ar.

**Figure 7:**
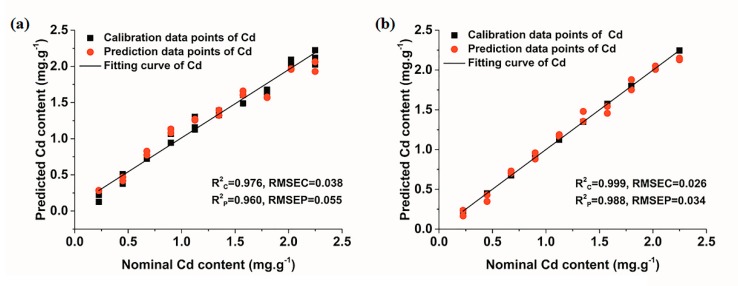
LS-SVM models of Cd under different conditions. (**a**) air; (**b**) Ar.

Figure 7 shows the results of LS-SVM models for the quantitative determination of Cd in soil. The good fitting result of all data points was obtained. R^2^ in the prediction set under both air and Ar conditions were greater than 0.96. In particular, the model performance under Ar condition was perfect, with R^2^ in calibration and prediction sets reaching 0.999 and 0.988, respectively. However, although the model under Ar condition performed well, it was only slightly better than that under air condition, which was similar to the results of PLSR models. As for the LOD of LS-SVM model, its calculation method remained to be further studied. On the whole, the results indicated that LIBS combined with the LS-SVM model had a reliable prediction power for quantitative analysis of Cd in soil. Moreover, signals collected in Ar condition were more suitable for the on-line detection of Cd content in soil with an improved accuracy.

#### 3.3.3. Comparison of Univariate and Multivariate Analysis Models

In order to evaluate the reliability of the detection performance of LIBS under both air and Ar conditions, we compared the results of three models, and they are summarized in Table 1. It was obvious that R^2^ of LS-SVM models under both air and Ar conditions were higher than that of the PLSR models and univariate models, and the trend was the opposite for the value of RMSEC and RMSEP. The LS-SVM model based on the spectra collected in the condition of Ar obtained the best result, with a high prediction accuracy close to one. As for the sensitivity of the model, compared with univariate models, Cd detection limits of PLSR models had been improved. In addition, the improving effect of Ar on the prediction accuracy of the univariate model was better than that of the other two models, indicating that Ar was more effective when the model had a relativity poor prediction performance. Therefore, it could be concluded that both chemometrics methods and signal enhancement techniques had influence on the quantitative detection performance of LIBS. Reasonable combination of chemometrics methods and signal enhancement techniques could greatly improve the LIBS quantitative detection capability. In this case, LIBS combined with the LS-SVM model and the signal enhancement technique of Ar realized fast and accurate detection of Cd content in soil. This work offered a potential method for the detection of heavy metals, and it could further provide guidance for environmental monitoring.

### 3.4. The Influence Principle of Ar on LIBS Analysis 

**Figure 8:**
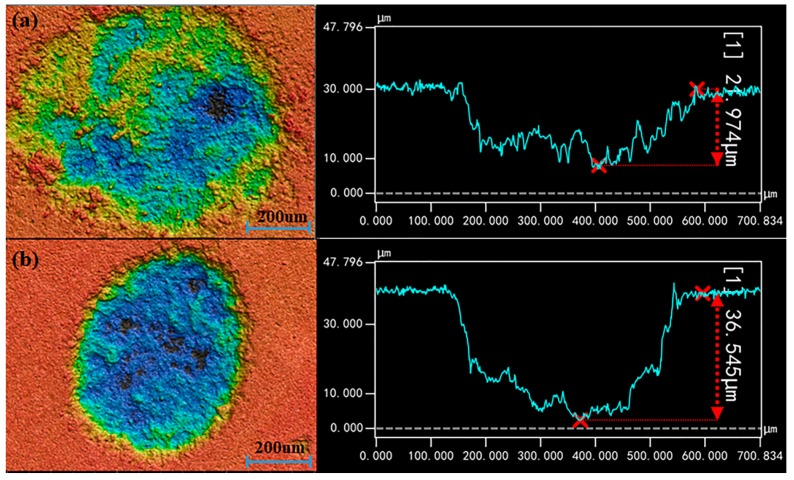
Comparison of the three-dimensional profile of the ablation crater. (**a**) air; (**b**) Ar.

Since LIBS elemental quantitative detection is based on the plasma generated from the laser ablation of samples, shapes of ablation craters were scanned by a three-dimensional contour scanner (VK-X-1000, Keyence, Osaka, Japan) to further analyze the effect of Ar on LIBS analysis. Multiple shots for each point of each sample were performed. It was found that the morphology of the ablation craters were approximately the same under the same experimental conditions, and the trend of parameters increasing with concentration were also consistent under Ar and air conditions. In order to explore the influence of Ar and be consistent with the above analysis, one three-dimensional profiles picture of the ablation crater in the soil sample with the content of 1.12 mg·g^−1^ under air and Ar conditions was taken as an example for comparative analysis, respectively, and the results are shown in Figure 8. Additionally, the average profile parameters of the ablation crater are listed in Table 2. Theoretically, the energy within the focused spot of the pulsed laser satisfies the Gaussian distribution. The energy density is the largest at the ablation center and gradually decreases toward the periphery [65]. Therefore, the target removal effect at the center of the ablation crater is the most obvious, and the overall appearance is a crater that is deepest at the center and get shallower outwards [66]. It was seen from Figure 8 that the morphology of ablated craters under both air and Ar conditions showed the state described above. However, they were not regular center deep shallow craters, and they also contained a variety of laser-induced micro-morphologies. It was also observed that the surface of the ablation craters produced many convex topographies, which was mainly due to the recoil effect of the target. When the target was severely heated by the laser radiation, the material near the ablation center would be lashed, and the backlash effect of the material made the surface of the ablation crater deformed [67]. As seen in Figure 8, the ablation craters under Ar conditions were deeper and the edges were smoother and more regular than those under air condition. This result was mainly credited to the flowing gas in the Ar condition, since a gas jet with a fixed flow rate could have produced a reproducible pressure field that overrode plasma fluctuations of the sample surface [29]. Thus, it could be concluded that the flowing Ar gas have effectively removed bubbles and make the surface of ablation crater smoother. From Table 2, the average volume, and maximum depth of ablation craters under Ar condition were larger than those under air condition, while the cross-sectional area was smaller than that in air. The results proved that sample ablation quality was higher under Ar condition, and it might be credited to the thermal properties of Ar. According to the Reference [28,68,69], the ionization potential of Ar is lower than that of air, so the ionization probability of Ar is higher than that of air. Thus, Ar is more easily ionized, resulting in greater electron density to promote plasma formation. Besides, the expansion of the plasma was limited under Ar condition due to the relatively large density of Ar. Moreover, the lower thermal conductivity of Ar caused high temperature of the generated plasma, leading to an increase in ablation quality [69]. In general, these three effects combined to make the LIBS signal stronger in Ar condition, which improved the quantitative analysis performance of Cd in soil.

## 4. Conclusions

This research revealed that LIBS combined with chemomentrics methods in Ar condition had a perfect ability to determine Cd content in soil. Several aspects including spectral intensity, System performance, quantitative analysis models of Cd under air and Ar conditions were compared. It was proved that LIBS in Ar condition acquired a higher spectral intensity and obtained a better system performance. Besides, the results of quantitative analysis of Cd in Ar condition were better than those in air condition. Moreover, LS-SVM models were superior to the other two models. Thus, the LS-SVM model in the condition of Ar showed perfect ability in the quantitative detection of Cd in soil, with R^2^ in both calibration and prediction sets more than 0.98. In addition, the influence principle of Ar on LIBS detection of Cd was analysed based on the three-dimensional profile of the ablation pit. It turned out that Ar could make laser focus more concentrated and ablate more quality of samples, which improved the LIBS signal intensity and quantitative detection ability. The overall results demonstrated that LIBS combined with the chemometrics method of LS-SVM in Ar condition could be a great tool for quantitative analysis of Cd in soil. With the perfect predictive performance, it was able to provide guidance for environmental monitoring. Additionally, portable instruments based on the method could be developed for further application.

## Figures and Tables

**Table 1:** Comparison of three chemometrics models of Cd under air and Ar conditions.

Data	Model	Parameter	R^2^_C_	RMSEC	R^2^_P_	RMSEP
	Univariate	-	0.862	0.101	0.849	0.105
Air	PLS-DA	3	0.959	0.053	0.940	0.065
	LS-SVM	(4.359 × 10^7^, 2.857 × 10^7^)	0.976	0.038	0.960	0.055
	Univariate	-	0.935	0.068	0.911	0.095
Ar	PLS-DA	2	0.981	0.035	0.973	0.051
	LS-SVM	(3.922 × 10^4^, 753.284)	0.999	0.026	0.988	0.034

Parameters of different models; the optimal number of latent variables for PLS-DA, the bandwidth of kernel function (sig2) and the trade-off between the minimum model complexity and the minimum training error (gam) for LS-SVM.

**Table 2:** Comparison of the average profile parameters of ablation craters under air and Ar conditions.

Condition	Volume/μm^3^	Cross-Sectional Area/μm^2^	Maximum Depth/μm
Air	2.15628 × 10^6^ ± 1 × 10	1.9626 × 10^5^ ± 1 × 10	22.0 ± 0.1
Ar	3.89284 × 10^6^ ± 2 × 10	1.5568 × 10^5^ ± 1 × 10	36.5 ± 0.2
